# Pyorrhœa Alveolaris—the Other View of It

**Published:** 1894-04

**Authors:** George S. Allan

**Affiliations:** New York


### PYORRHŒA ALVEOLARIS—THE OTHER VIEW OF IT.[1]

BY GEORGE S. ALLAN, D.D.S., NEW YORK.

Mr. Chairman and Members of the Odontological Society,—My excuse for being with you this evening seems to be twofold,—first, it was a pleasure to accept your kind invitation, and, secondly, I thought I had something to say worthy of your attention; but as to this last, the more I think over what has been said and written on my subject, the more doubtful I feel as to the wisdom of my coming. It is true I have some definite and settled views as to both the cause and the proper treatment of the so-called disease, pyorrhœa alveolaris, but I doubt much now whether I can impress you with the conviction that they are either new or specially valuable. But this I can say: They seem good to me. They help and guide me in my daily work, and I am convinced they look towards

---

[1] Read before the Odontological Society of Pennsylvania, February 10, 1894.

the truth. Then, in addition, my good friend Peirce has, in a measure, stirred up my combative disposition, and so I waive my scruples and misgivings, and bring you such food as I have for your mental repast this evening.

The hard and increasing work and study on pyorrhœa alveolaris is most encouraging, as to the results attained and to be attained in the treatment of this most troublesome disease. It is but a short time ago that the average practitioner wholly declined to even attempt its treatment, and advised his suffering patient to either have the offending member extracted at once or to suffer in silence, for he knew no means of alleviating the pain, much less of saving the tooth. The disease was akin to old age, and, like old age, was not amenable to treatment, and was equally hopeless. The *vis inertiæ* of utter ignorance as to the causation of the disease was too great to be easily overcome, and few dentists cared to waste either time or effort in the way of treatment. This happily is not now the case, and most of us are glad to be able to offer our patients some hope in the way of temporary, if not complete, salvation, and almost always are able to alleviate pain and discomfort.

Teeth are lost in one of two ways,—either through a destruction of their substance by decay or of their foundation; that is to say, they loosen in their sockets and fall out, or become so troublesome that they must be taken out. With caries, so-called decay of the teeth, we have nothing to do to-night.

The physical aspects or conditions found where the foundations of the teeth are destroyed vary very much, but for our purpose it is not necessary to enter into a close analysis or comparison of them. Leaving out of question what may well be called a senile loosening and consequent loss of the organs,—that which is solely a result of old age, where the gums and alveolar process disappear, waste away by absorption, exposing more and more, as time passes, the root, until at last the socket disappears, and the tooth is simply thrown off for want of support, a condition well understood and as hopeless for treatment as the age of the patient itself,—we have a variety of other conditions looking towards the same result if neglected, but which offer a fair field for investigation, study, and treatment, with the promise of more or less hopeful ending.

The day is past when the educated dentist can dismiss a patient with the statement that his disease is hopeless and that he will have to lose his teeth one by one as time goes on. For our purposes it would be a waste of time to go into any detail of the symptoms and conditions which mark the inception, progress, and ending of this disease, my object being only to draw attention to one or two special points. First let me say there is only one generic term I know of that will apply to all cases, the appropriateness of which cannot be questioned. The term is "luxation of the teeth." If to this generic term the two specific ones, pathogenic and traumatic, be added, we have a nomenclature that will fit all cases and conditions that present themselves. For it is true the presence of tartar in any of its varying forms is not essential to the disease, and the same can be said of pus. That, too, is often found wanting. An inflammatory condition in either the acute or subacute form may or may not be present, but the work of destruction goes on just the same. Necrosis of the processes is only an adjunct at times, and so we can go through the list. And it is not so rare a thing to find a case in which tartar, pus, inflammation and necrosis are all wanting, at least to all ocular evidence, and the disease still be present and progressive in its nature. In our present state of knowledge, it would be difficult to make strong assertions as to the rôle that germs play as a causative agent or agents. They, alas, seem to be omnipresent; but the amount of influence they exert for evil we have no present means of determining with exactness. One or more of the conditions named may be present, and some are much more common than others. They each and all are important or unimportant as the case may be. The fault we find with the classifications of Drs. Black and Peirce is that they do not recognize this fact.

Dr. Peirce's terms are but modifications of Dr. Black's, and by the introduction of the word "calcic" he recognizes the presence of tartar as the essential factor, the vice *par excellence*, to be sought for and eradicated, in combating the trouble. Dr. Black's terms, "serumic" and "phagedenic," go further, in that they recognize other factors besides the blood-serum and saliva as the sources of the active agents we seek for,—the power behind the throne. It appears to me very necessary that we should bear all these facts in mind if we want to be able to correctly diagnose and treat pathogenic or traumatic luxation of the teeth.

There may be said to be two camps of theorists in reference to this somewhat vexed question,—those who hold to the belief of its constitutional nature and those who refer it to a local exciting cause, and each side is quite sure it is right. A bias or preconceived opinion in any direction is hard to overcome. We are all more or less the slaves of our prejudices and opinions. There is a German school which takes the ground that all the diseases of the teeth are due to some constitutional vice, and as a consequence frown upon all local treatment, and take their pills, powders, and liquid medicines philosophically and in good faith. Now, I am one of those whose faith in the constitutional vice theory is very limited, and I feel quite certain that for all practical purposes the disease has a local origin, and that effective treatment must be based on this belief.

Therefore I look upon the paper which our able and scholarly colleague Dr. Peirce lately read before the New York Odontological Society as calculated to mislead and befog us, rather than to clear our vision and help us in a practical way. And yet no one of you more highly appreciates the great value of that paper, or more readily acknowledges the sincerity of purpose which pervades it, than myself. I confess also that I offer my criticisms upon it with many doubts and misgivings as to my ability to carry you with me.

First let me say that after hearing the paper read, and then carefully reading it in the January INTERNATIONAL DENTAL JOURNAL, I find but one new thought in it; or rather I should say, there is in it one old thought with a new prop or support to it. Briefly stated, it amounts to this: pyorrhœa alveolaris is a constitutional disease, and indicates a gouty diathesis, because a chemical examination of the deposits on the teeth shows the presence of free uric acid. The learned doctor, I think, has given us too broad a generalization from a limited amount of support. To properly and rightly present my arguments and conclusions, I quote from the printed paper.

"Specimen No. 1 contained, as shown by microscopical analysis, a number of fine needle-crystals of calcium urate, a few crystals of free uric acid, and crystals of calcium phosphate. Destructive distillation analysis yielded a strong ammonia reaction. The murexide test for uric acid and its compounds was faint, though the characteristic color showed in several places.

"Specimen No. 2 presented the same crystals on microscopical investigation. The murexide test was strong, producing a number of purplish-red spots.

"Specimen No. 3 yielded similar results.

"In addition to these three analyses by Professor Congdon, some six or eight specimens were examined by my friend and colleague Professor A. P. Brubaker, M.D., D.D.S., in my library and in my presence, the results obtained corresponding to those of Professor Congdon. In three of these an abundance of urate of soda crystals were observed. It must be remembered that, as the quantity embraced in each specimen was small in amount, large results could scarcely be obtained or expected."

Puzzling my mind over these analyses, those obtained by Dr. Peirce and those found in the books, the thought came into my mind that an independent one might help; so I interviewed my friend Professor Ricketts, of Columbia College, and obtained his assistance. Two samples, as his report shows, were given him. The one marked No. 1 was composed wholly of the black or serumic tartar from the roots of a number of teeth. The other, marked No. 2, was from the teeth of a patient having a strongly marked gouty and rheumatic condition of the system, partly an inheritance, partly acquired. The former was placed in the professor's hands, together with the published paper of Professor Peirce, with the request that he would make an analysis for comparison with those given by Dr. Peirce. The latter material was sent him just as it came from the teeth, with the request that he make a test for the presence of uric acid. Of course, this latter was not pure. It was mixed with the usual *débris* found in such positions and a large amount of salivary tartar. Still, there was a fair proportion of the black tartar present. With the above explanation, I now give you Professor Ricketts's report.

"CERTIFICATE OF ANALYSIS.

"NEW YORK, February 5, 1894.

"DEAR SIR,—The sample of tartar from teeth from ——, marked 'No. 1,' submitted for analysis, contains: Total solids, 63.80 per cent.; total organic matter, 36.20 per cent. The organic matter contains trace of uric acid."

"CERTIFICATE OF ANALYSIS.

"NEW YORK, February 5, 1894.

"DEAR SIR,—The sample of powder from ——, marked 'No. 2,' submitted for analysis, contains: Uric acid, none.

"Yours respectfully,

"RICKETTS & BANKS.

"To DR. GEO. S. ALLAN,
"51 West Thirty-seventh Street, New York."

These analyses prove, granting that they are accurate and contain no errors as to facts or conclusions, that a trace, and trace only, of free uric acid may be found at times in the black or serumic tartar commonly found clinging to the necks and roots of teeth in ordinary cases of pyorrhœa alveolaris. Dr. Peirce, I believe, claims more, but I question whether he can rightly do so.

The analysis (Dr. Peirce's) was qualitative only, and qualitative in respect to one constituent only. Now, at various times there have been quantitative analyses made of this same black deposit, and in no one of them do I find any reference made to the presence of uric acid. This fact alone would go to show that uric acid cannot enter largely into the composition of black tartar, for if it did it would certainly have been detected at an earlier date by some one of these chemists. Dr. Ricketts's was quantitative in part, so I take it for granted that we have to deal with a very small amount of uric acid,—so small that it would not seem to be safe or prudent to build so comprehensive a theory upon it, to the practical neglect of the other ingredients that go to make up the bulk of the deposit.

Again, free uric acid in the system does not necessarily prove a person to be either gouty or rheumatic. It is undoubtedly a symptom, and a strong one, but not a conclusive one. Free uric acid is frequently found in the blood-serum of persons who have neither a rheumatic nor a gouty tendency, and in these cases rather points to a condition of the system where destructive metabolism is in the ascendency. Waste is going on more rapidly than repair. Overwork, nervous or physical exhaustion are, as a rule, associated with this uric-acid condition of the system,—so much so that it is considered one of the most positive diagnostic symptoms of a run-down or enfeebled condition. A changed condition of life—one of rest, recreation, and enjoyment, followed by a building up of the system—will be followed almost immediately by the disappearance of the excess of waste products, including the uric acid and urates.

As further proof of the correctness of this position, I quote from an article in the *American Journal of the Medical Sciences* for February, 1891.

"V. Jaksh has examined the blood of patients in the effort to determine,—

"1. Whether uric acid took part in the acid intoxication occurring in febrile conditions.

"2. Whether it took part in the production of gout or attacks of gout.

"He refers to the statements which have already been made in support of this view, but says that investigations in the matter have never been carried out on a large number of patients.

"In all, he examined the blood of one hundred and five cases in a manner described by him. In nine cases of healthy individuals no uric acid was found. In nervous diseases it was also absent, and the same was true of nine cases of typhoid fever. In another case of this disease and in one of intermittent fever there was no uric acid present during the elevation of temperature, though it was detected after fever had disappeared.

"In the instances of diseases of the liver, stomach, and intestines examined, uric-acidæmia was only observed where anæmia was also present.

"The acid was found in diseases of the heart which produced cyanosis, as also in emphysema and pleural exudates. It was constantly present in considerable quantity in the febrile stage of five cases of pneumonia. In six cases of acute articular rheumatism there was no uric-acidæmia observed.

"Very considerable quantities of uric acid were found in all the cases of diseases of the kidneys, and in those of primary and secondary anæmias. These observations seem undoubtedly to prove that uric-acidæmia is not a pathognomonic symptom of gout."

Again, in "The System of Medicine," Pepper, article by Dr. W. H. Draper on gout, you will find this strong assertion:

"But apart from these physiological objections to the theory that uric acid is necessarily the offending substance in gout, it is well known that uric-acid salts accumulate in the blood in febrile diseases, in disorders of digestion, and in anæmia,—notably in splenic anæmia,—and do not produce either the symptoms or lesions of gout."

If Dr. Peirce is right, the presence or non-presence of pyorrhœa alveolaris would be a strong diagnostic symptom or the contrary of the gouty condition. But this is not the case; at least, physicians do not so consider it. In fact, there seems to be no recognizable connection between the two. For years I have been in the habit of asking patients coming for treatment if they were either gouty or rheumatic, and the answers, grouped together, have had only a negative value. I have been exceedingly anxious to find some constitutional vice that would bear the brunt of the trouble; and as it was in the air, as one might say, that gout and rheumatism were the suspected causes, I have seldom, if ever, neglected an opportunity of obtaining reliable working data to help in forming a correct diagnosis. I am sorry to have to report no satisfactory conclusions. A strong objection to the whole theory lies in the fact that, whereas depositions of tartar are so common as to be almost a rule in middle life and old age, I am thankful to say gout and rheumatism do not bear an undue proportion to the other ailments humanity labors with.

But let us, for the sake of argument, accept the conclusions the learned author arrives at, and see where they land us. Gout and rheumatism, especially the former,—or, to state the case in another way, all manifestations of either disease,—are followed by deposits of lime salts in the joints or other positions, and are deemed to be the most intractable of all diseases to treat. The deposit once formed, refuses to be dislodged. It cannot be reached. The blood-serum can carry fresh supplies of the objectionable material, but it cannot, no matter how it may be changed in character, redissolve it, take it back into the system, and dispose of it through any of the ordinary excretory channels. The same may be said of urinary calculi; and the poor mortal who has once passed one of these latter, no larger than a small pea, has had most painful evidence of the truth of this statement.

Now let me ask you the question in this latter case, Is the uric-acid state of the system the cause of the inflammation, pain, and distress, or is it the little lime nodule that happens to be situated where it is decidedly not wanted? How formed or whence came that nodule has nothing to do with the train of distressing conditions that it produces. It is a cause sufficient unto itself for them all. The vice in the system did form the nodule, but there its evil work ceased, and the nodule takes up the work where the vice leaves it. And as to these results, neither physician nor patient cares whether this nodule is composed of calcium phosphates, carbonates, or urates. They only want to get rid of it, and they work and labor only with that object in view, and they both know that when that is attained peace, comfort, and health return.

Now, I hold that the chemical constitution of this deposit has nothing to do with the train of evil results that follow its formation, though Dr. Peirce strongly intimates and accepts a contrary opinion. This you will see from the following quotation: "The fact, however, that in true pyorrhœa the symptoms are so different, and that all local treatment has been practically so unavailing, has suggested the possibility that some other chemical agent derived from the blood, and the product of some morbid constitutional state, might be the exciting cause," and he bases his argument strongly upon this belief. Now, it is most certainly true that no constitutional treatment can cause the blood-serum to take this deposit—let its composition be what it may—back into its current and carry it away. Professor Peirce says, "It is for this reason that I regard the deposition of uric acid as of blood origin, and the disease pyorrhœa alveolaris as one of the local manifestations of the constitutional state familiar to all pathologists as the uric-acid or gouty diathesis." If this statement were true, then the removal of the tartar and other local treatment would be unavailing, and a cure could not be effected, no matter how completely the parts were brought into a healthy condition. But this we know not to be the case. Cures are being constantly effected, though unaccompanied by constitutional treatment.

These reflections lead to one inevitable result. For all practical purposes, the constitutional-diathesis theory must be abandoned where treatment is to be adopted; and this I say fully impressed with the importance and value of the work done, and the facts brought to light as to the causation of the disease in what may be said to be the last analysis. Back of the local excitants and other factors of like nature that are the immediate cause of the disease, the wise man will seek means of prevention. But prevention and cure are two widely different things to think about, and call for as widely different means to attain the end sought for. Constitutional faults or vices do indeed lead to deposits of tartar, but when the tartar has been once deposited on the necks or roots of the teeth it starts in business on its own account, and on lines and in ways that its constitutional father never dreamt or thought of, and with results in the way of manufactured products that clearly indicate its ability to take care of itself. So by all means let the good work of the constitutional-diathesis theory workers be encouraged. The more they can do for us in the way of prevention the better; but we must not let them throw dust in our eyes, and lead us astray with the delusive hope that we can mend a broken head if we can only catch the boy who threw the stone and send him to the nearest Sunday-school.

It may be well to close with a few words in reference to treatment which appears to be most effective; and here let me say that, with many others, I hold that the present state of our knowledge does not permit of such a thing as a radical cure in any but a few exceptional cases, but I do most fully believe that much can be done, especially in the earlier stages of the disease, to ward off its evil effects, and even effect a permanent change for the better.

If my view be the correct one, these lesions and pathological conditions have their origin in some mechanical or chemical irritant obtaining a lodgement under the free margin of the gums, and to one holding this opinion it is not necessary either to underrate or deny the indirect influences that systemic cachexies have, either in aggravating or inviting them. Good judgment must be used in weighing the value and importance of each and all.

Looking at the condition of the teeth and adjacent soft tissues from a clinical stand-point, we find them much as follows:

1. As a rule, if the gingival borders have a healthy normal appearance and the teeth are not shaky, we may be assured that, though the disease has obtained a lodgement, it has attained no great headway.

2. Any departure from this condition shows progress in the disease, and that, too, just in proportion to the amount of the departure from the normal state. The thing to be done is to bring back the parts to the healthy condition, first, by removing the direct cause, and, secondly, by following the removal by local health-inducing treatment of the affected parts.

"Let it be recalled that in this form of the disease the morbid process begins at the root, and very frequently, if not usually, in the vicinity of the apical extremity."

This statement seems to be erroneous, and contrary to ordinary observed conditions. So far as my observation goes, the deposit is first formed just under the free margins of the gums, and in rare cases only at the apical ends of the roots, and I have yet to see a case where the so-called pocket did not point the way to reach it. Dr. Black, whose authority to speak is unquestioned, in the "American System of Dentistry," classifies pyorrhœa alveolaris, without question or exception, as a disease having its origin at the necks of the teeth; and if any other authority of note, excepting Dr. Peirce, does otherwise I do not know it. Kindly allow me time to use the black-board to illustrate my position, and I cannot do better than to make use of three marked cases that have come under my notice since deciding to come before you.

Now, a word as to how the deposit, where we have to deal with serumic tartar, is formed, and its nature. It is not easy to go back of the theory that the lime salts composing the deposit have been carried to the parts through the medium of the blood-serum, but it is no slight problem to indicate how it has been accomplished. Possibly the principle of diffusion may account for it, but it cannot be so stated positively. In the large majority of cases the deposit in the early stages will be found just under the free margin of the gums and above the peridental membrane, and this would seem to show that it could not have been directly carried to the parts by the blood current. In some way the plasma, weighted with the salts, is exuded or pressed through the membranes, and attaches itself to the necks of the teeth. The formation of tartar deposits is a gradual one, and as it goes on it is brought more or less under the influence of the saliva and other fluids in the mouth; to what extent these may modify or change its nature we can hardly guess. But the probabilities are that they do affect the characteristics, physical and chemical, of the deposit to a considerable degree, and there is little doubt that the *débris* of the mouth also forms a part of the deposit. Microscopical examinations indicate this very strongly. I am not in a position to question the judgment of Dr. Peirce where he says, "As the current of the lymph-stream is directed for the most part towards the cementum, through its borders or periphery into the lacunæ and canaliculi, and finally in the reverse direction, it is not difficult to see why the deposit should take place on the surface of the cementum as well as in the meshes of the alveolo-cemental membrane." But I confess I do not quite understand what Dr. Peirce means by his reference to the lymph-stream; but it seems to me that proof positive that it is so formed and deposited is wanting, other than it may be in a few exceptional cases. The deposit as found is amorphous; no crystals ever go to form any part of it, and it is not constant, so far as we know at present, either in its chemical or physical characteristics.

The ways of removing deposits on the teeth are at our command,—the mechanical and the chemical. As to the first, it is mostly a matter of skill, controlled by the educated eye and touch. Force to any amount is seldom required, and never to the extent of injuring the soft tissues or the alveolar processes. Dr. Van Woert, whose most excellent paper on this same subject may be found in the January, 1894, number of the INTERNATIONAL DENTAL JOURNAL, is wholly right in what he says on this point. The chemical agent I mostly rely upon is sulphuric acid, using a ten-per-cent. solution of the pure manufacture.

One point I would lay special stress upon, in closing, is the vital necessity where teeth have become loosened in their sockets in some way to give them support. The constant motion of a shaky tooth of itself rapidly destroys what foundation is left, and all work is useless that does not contemplate holding the teeth firmly in place as of the first importance.

